# Shark preferences: Only subtle effects of electromagnetic field exposure and substrate type on small‐spotted catshark (*Scyliorhinus canicula*) behaviour

**DOI:** 10.1111/jfb.70583

**Published:** 2026-07-28

**Authors:** Lotte J. Bouwman, Annemiek Hermans, Jeroen Hubert, Reindert Nijland, Henrice Jansen, Jildou Schotanus, Hendrik V. Winter

**Affiliations:** ^1^ Aquaculture & Fisheries Group Wageningen University and Research Wageningen The Netherlands; ^2^ Wageningen Marine Research Wageningen University and Research IJmuiden The Netherlands; ^3^ Marine Animal Ecology Group Wageningen University Wageningen The Netherlands

**Keywords:** behaviour, benthic shark, impact assessment, mussel substrate, offshore wind, subsea power cable

## Abstract

Offshore wind energy plays a crucial role in the transition to low‐carbon power generation. The expansion of offshore wind farms (OWFs) introduces anthropogenic electromagnetic fields (EMFs) from subsea power cables. Elasmobranchs, including sharks and rays, are sensitive to natural electric and magnetic fields used for navigation and prey detection, making them potentially vulnerable to anthropogenic EMFs as they may alter orientation, foraging or habitat use. Coastal shelf seas, where most OWFs are constructed, often overlap with essential elasmobranch habitats, including complex biogenic structures such as mussel beds that provide foraging and refuge opportunities. We aimed to determine whether exposure to an AC‐EMF representative of subsea power cables and/or complex mussel substrate over non‐complex substrate influences behaviour and substrate preferences in juvenile small‐spotted catsharks (*Scyliorhinus canicula*). This study investigated the behavioural responses of 14 juvenile *S. canicula* exposed to a gradient of alternating current (AC) EMF (max. RMS 2 μT) and complex biogenic mussel substrate in a 2^2^ factorial design in a circular preference chamber. Across 28 trials, time spent and distance travelled in each quadrant did not differ with EMF exposure and/or the presence of complex biogenic substrate. Hidden Markov models revealed subtle but significant changes in behavioural states (inactive, local activity, transit), where sharks were more inactive (+6%pt.) and less locally active (−5%pt.) in the EMF + mussel quadrant compared to the bare and EMF‐only quadrants, and increased transiting behaviour (+6%pt.) in the mussel‐only quadrant relative to the EMF + mussel quadrant. These findings show that *S. canicula* juveniles show no preference in substrate or alternating‐current EMF exposure, but they did subtly change their behaviour. Future work should examine direct‐current systems and field‐based tracking and assess the extent to which these observations generalise to field settings and other elasmobranchs.

## 1 INTRODUCTION

### 1.1 Electromagnetic fields from offshore generated energy transport

Offshore wind farms represent a sustainable and expanding source of energy for countries bordering the sea (Díaz & Guedes Soares, 2020; Isaksson et al., 2025). As part of offshore infrastructure, subsea power cables are required to transport electricity from turbines to high‐voltage offshore substations and subsequently to shore via export cables (Henkel et al., 2014; Taormina et al., 2018; Worzyk, 2009). These cables generate electromagnetic fields (EMFs), introducing anthropogenic EMFs into the marine environment (Garavelli et al., 2024; Henkel et al., 2014; Taormina et al., 2018). Naturally occurring EMFs in the ocean arise from the earth's geomagnetic field, ocean currents, and geomorphological features, and bioelectric fields produced by marine organisms (CSA Ocean Sciences Inc. and Exponen, 2019; Gill et al., 2014; Hutchison, Gill, et al., 2020). Natural EMFs are used by different marine taxa for ecological purposes including navigation, foraging, and interaction with conspecifics (Albert et al., 2020; Hermans et al., 2024; Verhelst et al., 2025). Despite increasing awareness, the effects of anthropogenic EMFs on marine species remain poorly understood (Garavelli et al., 2024; Hermans et al., 2024; Hutchison & Gill, 2025; Verhelst et al., 2025). With the predicted expansion of offshore wind farms, creating a reticulated EMF network across benthic habitats (Hermans et al., 2024). However, knowledge gaps remain regarding the behavioural consequences for elasmobranch populations of such exposure, particularly in relation to benthic habitats common in offshore wind farms.

### 1.2 Elasmobranch and EMFs

Elasmobranchs, including sharks, are capable of detecting natural electric and magnetic fields, making them potentially susceptible to changes in EMFs (Kalmijn, 1971; Kalmijn, 1982; Meyer et al., 2005). As ecologically important predators, increasingly threatened by human activities, understanding factors that may affect their behaviour and habitat use is essential to understand the potential impact on these species (Dedman et al., 2024; Dulvy et al., 2021; Flowers et al., 2021). Elasmobranchs detect electric fields primarily through specialised sensory organs known as the Ampullae of Lorenzini, which are distributed over the head and pectoral region (Newton et al., 2019; Sisneros & Tricas, 2000). There is less consensus on how magnetic fields are sensed, but this is hypothesised to occur either indirectly through the same organs that detect electric fields or via magnetite‐based receptors, enabling navigation over large distances (Crampton, 2019; Nyqvist et al., 2020). Electro‐ and magneto‐sensitivity play critical roles in foraging (Bedore & Kajiura, 2013; Kajiura & Holland, 2002), mating interactions (Kempster, Garza‐Gisholt, et al., 2013; Sisneros & Tricas, 2000; Tricas et al., 1995) and navigation (Anderson et al., 2017; Kalmijn, 1978; Keller et al., 2021; Naisbett‐Jones & Lohmann, 2022). These senses also aid in predator avoidance during embryogenesis, as the embryo senses a predator and responds by ceasing all movement, reducing the chance of detection (Ball et al., 2016; Christensen et al., 2023; Hermans, Maas, et al., 2025; Kempster, Hart, & Collin, 2013; Paoletti et al., 2026), underscoring their importance for survival. Anthropogenic EMFs generated by subsea power cables may interfere with these natural cues, potentially altering habitat selection or foraging efficiency (Hermans et al., 2024; Hutchison, Secor, & Gill, 2020). To investigate these potential effects, we focus on the small‐spotted catshark (*Scyliorhinus canicula*) (Linnaeus, 1758), a species well‐suited for controlled experiments due to its small size, tolerance of captivity, relatively high abundance and ex‐situ availability (Compagno et al., 2005; Ellis et al., 2008; Heessen et al., 2015). Juveniles were selected as relevant study subjects, as early developmental stages are often more sensitive to environmental perturbations (Ball et al., 2016; Hermans et al., 2024; Hermans, Maas, et al., 2025; Wheeler, 2023), and impacts during this period may carry over to later life stages.

### 1.3 Potential impact of EMFs on habitat preference

Benthic sharks such as *S. canicula* commonly inhabit shallow coastal environments, including sandy areas, rocky substrates and biogenic reefs formed by *Sabellaria* worms, mussels or other shellfish, which provide both shelter and foraging opportunities (Heessen et al., 2015; Martin et al., 2012; Papadopoulo et al., 2023; Pizzolla, 2025). Mussels are ecosystem engineers that represent a structurally complex substrate that supports diverse benthic prey communities and prevail in deltaic regions (Buschbaum et al., 2009), where subsea cable routes are frequent. Mussels also colonise artificial structures, including platforms and potentially scour protection around offshore wind farms (Degraer et al., 2020; Maar et al., 2009; Ter Hofstede et al., 2022). Moreover, mussel aquaculture is globally widespread in soft‐sediment coastal systems (Ysebaert et al., 2009).

The diet of *S. canicula* includes a wide variety of benthic organisms, including species associated with mussel presence, but commonly not mussels directly (Kousteni et al., 2017; Pizzolla, 2025). If anthropogenic EMFs alter habitat preferences, this could render large areas less suitable for sensitive species through deterrence or conversely attract individuals potentially reducing energy expenditure efficiency (Hutchison, Secor, & Gill, 2020; Labourgade et al., 2024; Taormina et al., 2018). There are studies that indicate such attraction (Gill, 2009; Hutchison, Gill, et al., 2020; Labourgade et al., 2024). Most critically, EMFs may interfere with prey detection, for example by masking bioelectric cues, thereby disrupting foraging patterns and effectively reducing suitable habitat for species already under ecological pressure (Hermans et al., 2024; Hutchison, Secor, & Gill, 2020; Newton et al., 2019; Taormina et al., 2018). Over 5.5% of the Dutch continental shelf, an important habitat for this species, is projected to be exposed by anthropogenic EMF by 2030 in a high wind scenario. Understanding these dynamics is essential to assess offshore wind impacts, optimise cable routing and identify potential mitigation needs.

### 1.4 Aim and approach

We aimed to determine whether juvenile *S. canicula* prefer complex substrate over bare substrate, and whether exposure to an EMF representative of exposure at 1 m (minimum burial depth) of a 700 MW subsea power cable modifies their substrate choice and behaviour under controlled laboratory conditions. To address this objective, we employed a circular preference chamber divided into four quadrants representing EMF‐ and complex substrate presence/absence in a 2^2^ factorial design. As a proxy for ecologically relevant and complex substrate, the chamber bottom was covered with live blue mussels (*Mytilus edulis*) for two quadrants as against two quadrants with bare bottom. A local electromagnetic field, with EMF values comparable to those on a seafloor generated by subsea power cables, was generated using a solenoid. This design allowed us to disentangle the effects of EMF exposure and presence of substrate structure on substrate preference and shark behaviour. The documented attraction to anthropogenic EMF (Gill, 2009; Hutchison, Gill, et al., 2020; Labourgade et al., 2024) and the perceived preference to biogenic reefs (Heessen et al., 2015; Martin et al., 2012; Papadopoulo et al., 2023; Pizzolla, 2025) lead to the hypothesis that EMF and substrate complexity influence behaviour and spatial distribution of juvenile *S. canicula*.

## 2 MATERIALS AND METHODS

### 2.1 Test animals

Fourteen juvenile *S. canicula* of 6–9 months old were obtained from Wageningen University and transported to the WUR Yerseke facility. During transport, sharks were placed in plastic bags containing ∼10 L seawater and filled with 2/3rd 100% oxygen and transported in Styrofoam boxes to maintain temperature. The transport duration was approximately 120 min. At the start of the experiment in May 2025, the juveniles were photographed for individual ID, weighed and assessed to be in good health (e.g., no ectoparasite presence, abrasions or lesions, good overall condition). Half of the individuals used in this experiment were not naïve to anthropogenic AC EMF, as they were chronically exposed to low‐level AC EMF (1.8–4.6 μT) during embryogenesis. All individuals were exposed 2 times to a brief 10 min EMF stimulus (1 μT and 10 μT) at 5 and 11 weeks respectively. The behaviour and biometric parameters were assessed at week 5, 8 and 11 after hatching, and no significant changes in baseline behaviour were found resulting from the EMF exposure during embryogenesis (Hermans, Hubert, et al., 2025). To ensure conscious and responsible use of experimental animals, sharks were considered naïve again after a period of 3 months without further exposure. Although this period was considered sufficient to minimise short‐term carry‐over effects, prior exposure history is acknowledged as a potential source of variation.

### 2.2 Husbandry

Sharks were housed in a sound‐ and temperature‐isolated climate room (Wageningen Marine Research, Yerseke, Zeeland, The Netherlands) that was temperature‐controlled at ±16°C, corresponding to the outside temperature at the time of the experiment. A 12:12 h light: dark photoperiod was automatically maintained, with dim light (5 lx; Lutron LX‐107, sensitivity = ± 0.05%) from 7 am to 7 pm for simulation of underwater light conditions. To familiarise the sharks and mimic a more varied environment, ~20% of the tank bottom was covered with live mussels. Sharks were group‐housed in a 470 L light‐grey‐coloured propylene housing tank measuring 119 × 79 × 75 cm (LxWxH) subdivided into two sections using a propylene grid, with individuals moved to the other section after completing their initial trial. During the day, a constant inflow (3 L per minute) of ambient temperature seawater was pumped from the Eastern Scheldt, passed through a coarse sand filter, into the housing tank and aerated with air stones to maintain stable oxygen levels. Water quality was assessed daily at noon (portable multiparameter, HANNA HI98194) for temperature (17.21°C ± 1.11 SD), pH (7.98 ± 0.14 SD), dissolved oxygen (74.69% ± 12.58 SD) and salinity (27.46 ppm ± 0.71 SD). Sharks were fed ad libitum, with a minimum of 3–4 cubes (~0.5 cm^3^ each) of fresh whiting (*Merlangius merlangus*) and pouting (*Trisopterus luscus*) daily (visually checked), equalling ~2.5% of wet body mass. Additional feed was placed on the tank floor, ensuring that feed remained available for consumption at a later stage. Leftover food was removed the next day, prior to feeding. During feeding, visual welfare checks confirmed active swimming and feeding behaviour of each individual. Maximum background EMF levels in the housing tank were 0.13 μT ± 0.06 sd (Narda ELT‐400 magnetometer).

### 2.3 Experimental set‐up

A sand‐coloured polypropylene circular preference chamber with a 190 cm diameter (Dye et al., 2024; Post et al., 2017; Schram et al., 2013) was used to assess the potential influence of electromagnetic fields (EMF) and habitat preference of juvenile sharks. The chamber design was adapted from Myrick et al. (2004) and has been used in previous behavioural studies with different fish species (Dye et al., 2024; Post et al., 2017; Schram et al., 2013). The chamber was comprised of a central compartment and three rings: a mixing channel; a swimming channel and an effluent channel. Sharks were placed in the swimming channel with a width of 19 cm, in which the sharks could move freely. Water entered the mixing channel at eight evenly spaced inlets, passing evenly through circular apertures into the swimming channel and then the effluent ring, draining via the central section to five 60‐L storage tanks beneath. More detailed descriptions can be found in Dye et al. (2024) and Schram et al. (2013).

The circular chamber was placed in the same climate room as the housing tank, under the same light‐ and temperature conditions. In the chamber, the swimming channel was equally divided into four quadrants of a 90‐degree angle. Each of the four quadrants contained a different experimental condition: (1) bare substrate without EMF exposure, (2) bare substrate with EMF exposure, (3) mussel substrate with EMF exposure and (4) mussel substrate without EMF exposure. This design enabled direct comparison of habitat use between complex (mussel) and non‐complex (bare) substrates under both EMF and non‐EMF conditions (Figure 1). As a proxy for complex biogenic substrate, live mussels forming a 3D structure on the bottom of the swimming channel were used. For each trial, 1–1.2 kg of live blue mussels (*Mytilus edulis*) originating from the Eastern Scheldt were used, forming a bed of mussels in the complex substrate condition quadrants. Before use, epifauna were removed and they were rewatered for at least 12 h to filter out sand to eliminate any confounding factors. Four overhead cameras (Basler 1300‐60gm; monochrome; 1.3 Mp; 60 fps) paired with four infrared led‐lights (Raytec VAR2‐i2‐1; IR 850 nm) were positioned above the chamber to record shark swimming patterns during light and dark conditions.

**FIGURE 1:**
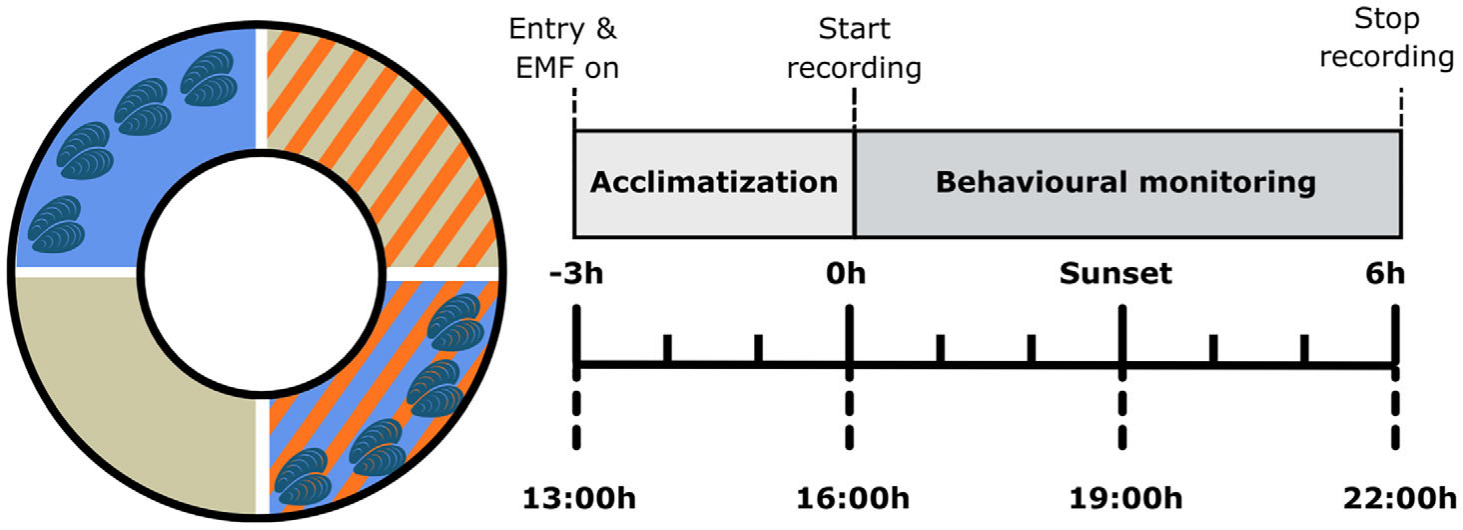
(a) Schematic overview of the habitat preference chamber indicating the different conditions per quadrant, where blue indicates mussels and orange stripes indicate exposure of electromagnetic fields. (b) Overview of the time schedule used during the trials. Time of entry was at 13h00m, 3 h before sunset, after which sharks were given 3 h to acclimatise to the new environment. The trial lasted 6 h and finished 3 h after sunset to capture behavioural changes in both light and dark conditions. Image created with https://BioRender.com.

During the experiments, seawater from five 60‐L storage tanks underneath the chamber was circulated continuously through the chamber via eight inlet points. A constant inflow of 3 L of ambient temperature seawater per minute was maintained for the chamber, administered in the storage tanks. Directly before and after each trial, water quality was recorded for every quartile of the circular chamber, resulting in averaged water temperature (17.6°C ± 1.2 SD), pH (8.0 ± 0.1 SD), dissolved oxygen (5.55 mg/L ± 0.97 SD) and salinity (34.1 ppt ± 0.7 SD) values across all trials (portable multiparameter, HANNA HI98194). At the end of each trial, after water quality measurements, all water in the preference chamber was refreshed to remove any chemical cues from the previous individual.

The EMF was generated by a solenoid fixed to a high‐density polyethylene (HDPE) frame connected to an Accel Instruments TS250 Waveform Amplifier, set to Hi‐Z and 20 dB gain (hereafter referred to as the EMF coil). The solenoid, with a diameter of 800 mm and 155 turns of 1.5 mm^2^ copper wire, was paired with a fixed one‐axis fluxgate magnetometer positioned 30 cm behind it. All components were custom‐designed and integrated by Waterproof Marine Consultancy & Services BV. A 50 Hz AC magnetic field was generated, creating a localised EMF gradient across the experimental arena. Field strength was monitored using a fixed single‐axis fluxgate magnetometer positioned in the centre of the solenoid and verified before and after each trial using a Narda ELT‐400 magnetometer at fixed positions within each quadrant. Reported EMF values represent RMS magnetic flux density in μT. Background AC EMF levels were elevated relative to natural conditions originating from the interfering structural elements of experimental room which could not be removed (0.32 ± 0.15), yet remained stable at over six times lower than the exposure condition. Directly before and after each trial, the highest observed EMF values were recorded at four equally spaced positions per quadrant, to determine if the exposure values were as planned. For trials 1–4, the EMF level of only one position per quadrant was measured, and therefore linearly interpolated for positions in between. From trial 5 onwards, four EMF values for every quadrant were recorded (16 positions in total), increasing the resolution of the EMF gradient across the chamber. Measured just before and after all 28 trials, the average recorded EMF values in the swimming channel were: 2.04 μT ± 0.5 SD in front of the EMF coil; 0.49 μT ± 0.43 SD in the edge zones of the EMF field and 0.32 μT ± 0.15 SD in the position opposite the EMF coil (bare/bare + mussel condition).

### 2.4 Experimental protocol

The EMF coil and substrate were positioned and the field was activated before the sharks entered the preference chamber, to prevent any novelty effects (onset response) associated with EMF exposure or mussels. Sharks were tested individually to eliminate behavioural influences from conspecifics. For each trial, an individual was randomly selected from the housing tank and placed into one of the eight zones (at random) of the circular chamber between 12:00 and 13:00 (Table S3). Data collection started only after 3 h to minimise behavioural effects associated with environmental acclimation and recovery from handling. Handling behaviours were observed as moments of surface bobbing and elevated swimming speed, for which the duration varied between individuals from at least 5 min to up to 2 h after entering the preference chamber. Hence, between two to 3 h, no novelty‐ nor handling effects were observed anymore and sharks resumed usual behaviour. Swimming behaviour was recorded for 6 h—from 3 h before to 3 h after artificial sunset at 19:00 (i.e., 16:00–22:00)—to capture light–dark changes in activity. Each individual completed two trials, separated by at least 3 days to reduce effects of habituation and carry‐over effects, resulting in a total of 28 trials.

The EMF coil location was randomly assigned to one of five possible positions, locations adjacent to the housing tank were excluded to avoid exposing stock fish to EMF (Figure 5). The location of the mussel condition was also randomly assigned, with the second mussel condition quadrant placed opposite the first to ensure clear choice opportunities.

EthovisionXT v.17.5 (Noldus et al., 2001) was used to record swimming patterns, yielding an XY position every 0.2 s for subsequent analyses. Movements of less than 0.5 cm were ignored to prevent detection interference by mussels (Ethovision setting: track smoothing). Tracking was conducted independently per camera, producing four XY coordinate streams that were subsequently merged for each trial. Positional data were then corrected to match arena dimensions and the randomised experimental conditions per quadrant for each trial. This process produced a continuous XY position dataset for each shark, annotated with the corresponding experimental condition at each time step. For both trials 5 and 16, the last hour of XY datapoints were excluded due to a recording problem in EthovisionXT. To account for this reduced recording time, behavioural metrics were scaled to a standardised 6‐h duration prior to analysis.

### 2.5 Statistical analyses

To check whether a sharks' release location, for example, condition upon entering the preference chamber influenced the number of minutes spent per condition, a Kruskal–Wallis rank sum test was performed. Quadrant use was determined by comparing the time spent and the distance travelled between quadrants, both for all trials (*n* = 28), and averaged/summed per individual (*n* = 14). When comparing quadrants across trials, the trials were considered repeated measures by individual shark, whereas data was considered independent when comparing across individuals. Prior to performing statistical comparisons, data were inspected for normality and homogeneity of variances using standard diagnostic tests (Shapiro and Levene's test). Because assumptions for parametric testing were not met, all subsequent analyses were performed using the non‐parametric Friedman rank sum test (comparing across trials) and Kruskal‐Wallis rank sum tests (comparing across sharks). Analyses were performed using R Statistical Software (v4.4.1; R Core Team 2025).

Next, we analysed behavioural dynamics using discrete‐time hidden Markov models (HMMs). Since behaviour cannot be measured directly from the swimming pattern data, the HMMs estimates the most likely underlying behavioural state from the observed time series data. The HMMs were run in R using the package ‘momentuHMM’ (version 1.5.6; McClintock & Michelot, 2018). The sharks' horizontal step length (swimming speed) (Figure 2a) and turning angle (Figure 2b) were used as input data for model fitting. These two metrics are commonly used in such movement HMMs because they reflect complementary aspects of behaviour. To identify the number of behavioural states best supported by the input data, null models with one up to three states were compared using AIC scores. We used three states a maximum because model selection criteria applied to HMMs often favour models with more states than biologically interpretable (cf Pohle et al., 2017). The three‐state model had the best fit (Table S1) and we labelled the states as (1) inactive—low step length and an uniform distribution of turning angles, (2) locally active—moderate step length and a low directionality and (3) transiting—large step lengths and highly directional movement (Figure 2).

**FIGURE 2:**
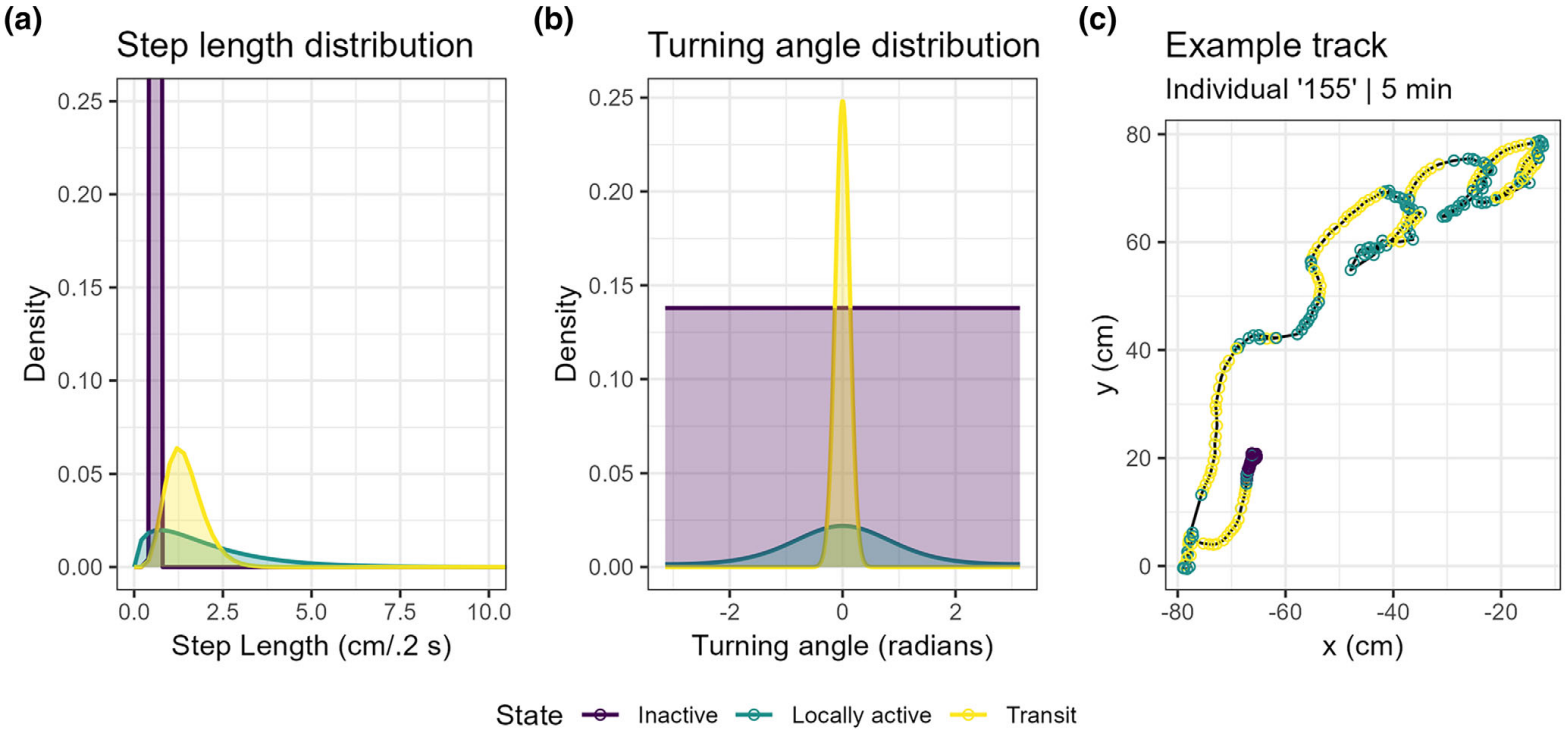
Distribution of step length (a) and turning angle (b) of the three behavioural states (colours) as classified by the hidden Markov model (HMM). (c) Top view of a 5 min swimming track, with the classified behavioural states.

Combinations of the covariates: quartile (bare, EMF, EMF + mussels, mussels), order, body length and light condition (light, dark) were included as predictors of state transition probabilities. By including covariates, they influence the probability of switching from one behavioural state to another, allowing us to quantify how experimental or other factors shape behavioural dynamics. Forward model selection based on AIC comparison indicated that the model including all covariates provided the best fit (Table S2). The significance of covariate effects on state transition probabilities was assessed using likelihood ratio tests (LRTs) comparing models with and without the focal covariate. For the best‐fitting model, stationary state probabilities (i.e., the modelled proportion of time an individual would spend in each state under constant covariate conditions) were calculated with 95% confidence intervals and plotted to visualise the size and direction of covariate effects. These probabilities describe the stable proportion of time an animal would spend in each state if the covariates remained constant, integrating the full set of transition probabilities into a single interpretable measure.

## 3 RESULTS

A total of 26 6‐h trials and two 5‐h trials were conducted using 14 juvenile *S. canicula* to assess differences in preference and behaviour between four quartiles with(out) EMF exposure and with(out) complex biogenic substrate. All 14 individuals appeared healthy from the beginning to the end of the study.

### 3.1 Habitat preferences

All individuals actively moved around in the circular preference chamber during at least one of their two trials (Figure 5). When visualising the swimming tracks and position of the EMF for each trial (Figure 5), release location appears to not affect where sharks spent the greatest proportion of time based on visual evaluation. Correspondingly, no association between release location and time spent per condition was observed (Kruskal–Wallis rank sum, *p* = 0.5102, $\chi_{3}^{2}$ = 2.3123). No abnormal or stress‐related behaviours such as belly swimming or surface bobbing were observed during the trials. No statistically significant differences were found between the number of minutes spent across the four quadrants with different experimental conditions (1) bare substrate without EMF exposure, (2) bare substrate with EMF exposure, (3) mussel substrate with EMF exposure and (4) mussel substrate without EMF exposure across trials (Friedman rank sum test, *p* = 0.8839, $\chi_{3}^{2}$ = 0.65414) nor distance travelled between condition across trials (Friedman rank sum test, *p* = 0.4057, $\chi_{3}^{2}$ = 2.9098). Similarly, no significant difference was observed in the number of minutes spent between conditions across sharks (Kruskal–Wallis rank sum, *p* = 0.4129, $\chi_{3}^{2}$ = 2.8652, Figure 3), nor in total distance travelled between conditions across sharks (Kruskal‐Wallis rank sum, *p* = 0.713, $\chi_{3}^{2}$ = 1.367347). Overall, these results indicate that neither EMF exposure nor presence of mussels influenced the quadrant selection of juvenile sharks in this experiment.

**FIGURE 3:**
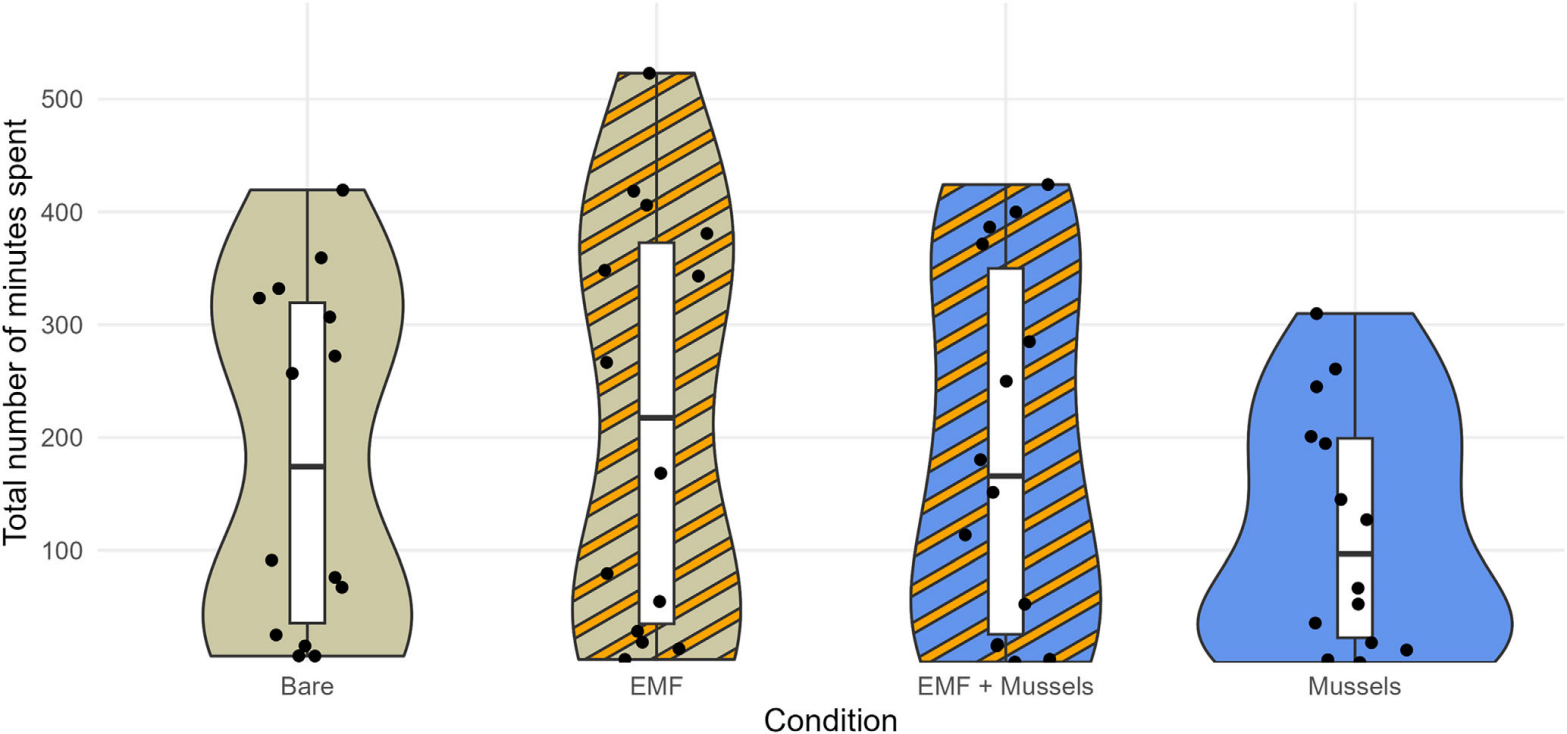
Time spent per individual shark (minutes; *n* = 14) at four quadrants, each representing a distinct experimental condition (1) bare substrate without EMF exposure, (2) bare substrate with EMF exposure, (3) mussel substrate with EMF exposure and (4) mussel substrate without EMF exposure. For each shark, data were summed across two 6‐h trials, with trials 5 and 16 corrected for their 5‐h recording only. Violin plots depict the distribution across individuals; inset boxplots show the median and interquartile range. The number of minutes spent did not differ significantly among conditions (Kruskal–Wallis rank sum, *p* = 0.4129, $\chi_{3}^{2}$ = 2.8652).

### 3.2 Behavioural changes

On average, sharks were inactive for approximately 85% of the trial, with the remaining time roughly evenly divided between transiting and locally active (Figure 5). Individual variation was high in both overall activity and quadrant use (Figure 5; Figure S1), and several individuals showed different behaviour between trials (e.g., individuals 186 and 25).

The quartile (bare, EMF, EMF + Mussels, Mussels) had a significant effect on behavioural state transition probabilities (LRT: $\chi_{18}^{2}$ = 1381.3, *p* < 0.001). Differences in stationary state probabilities among quartiles are generally small, with sharks being more inactive (up to 6%pt.) and less locally active (up to 5%pt.) in the EMF + Mussels quartile when compared to the bare and EMF quartiles, and transiting more (6%pt.) in the Mussels quartile than in the EMF + Mussels quartile, as indicated by non‐overlapping 95% confidence (Figure 4). State transitions were significantly influenced by diel light conditions (LRT: $\chi_{6}^{2}$ = 849.3, *p* < 0.001), with both locally active and transiting increasing during dark hours, reducing inactivity with ~20%pt (Figure S2). Trial order also had a significant effect (LRT: $\chi_{6}^{2}$ = 7750.2, *p* < 0.001), with sharks being slightly less likely to move (~3%pt.) in the second trial (Figure S3). Additionally, body length had a significant effect on state transition probabilities (LRT: $\chi_{6}^{2}$ = 593.1, *p* < 0.001) with larger individuals being more inactive than smaller sharks (Figure S4).

**FIGURE 4:**
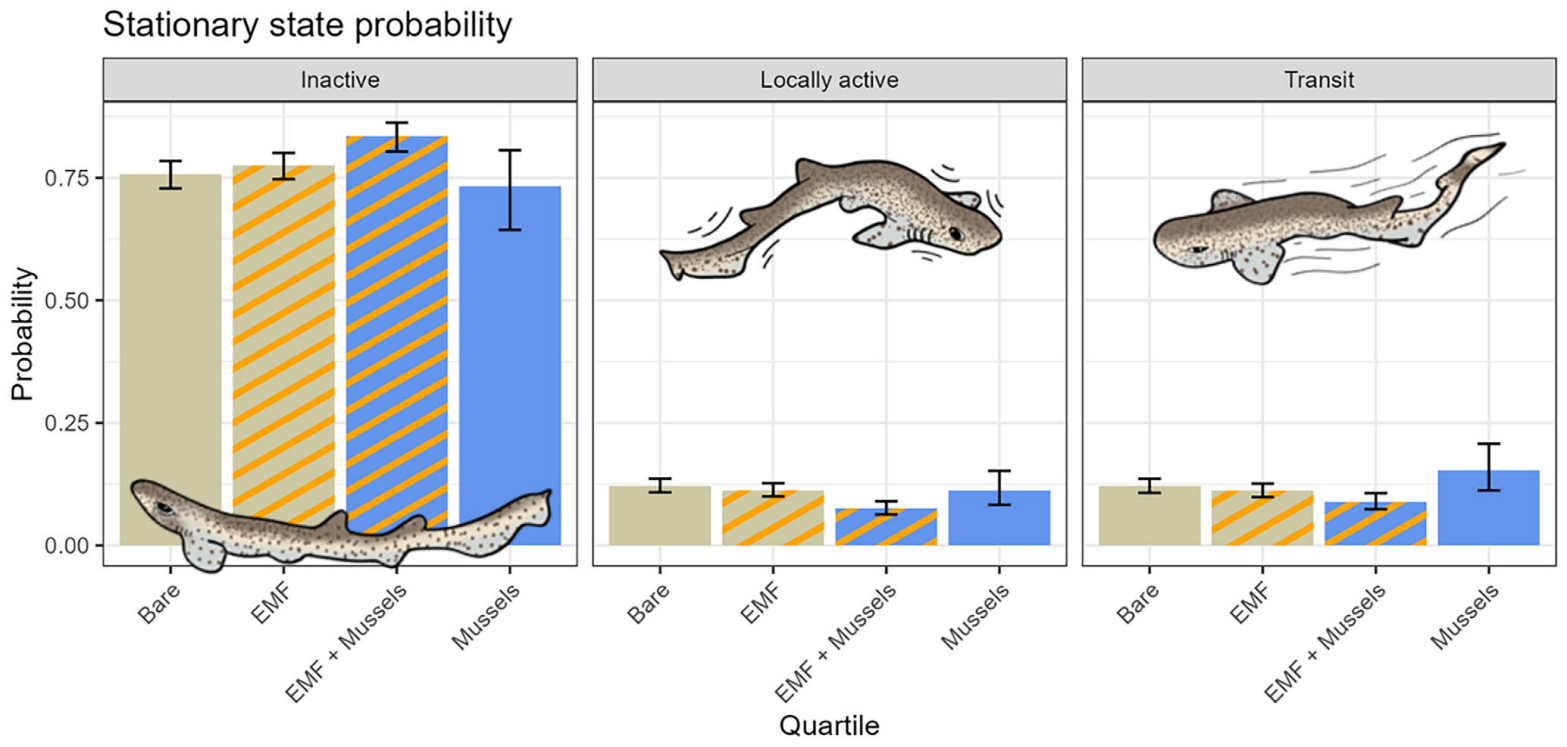
The stationary state probability of the three behavioural states of the 14 sharks, for each condition (1) bare substrate without EMF exposure, (2) EMF exposure with bare substrate, (3) EMF exposure with mussel substrate and (4) mussel substrate without EMF exposure. The error bars represent the 95% confidence interval. In the EMF exposure with mussel substrate condition, the sharks spent more time being inactive than in the EMF exposure with bare substrate.

## 4 DISCUSSION

Substrate preference and swimming behaviour of juvenile *S. canicula* exposed to SPC‐related EMFs and two substrate types were assessed using a controlled preference experiment. No differences in overall space use were detected among quadrants representing the different experimental conditions. However, sharks exhibited subtle but significant shifts in behavioural state transition probabilities, with increased inactivity (+6%pt.) and reduced local activity (−5%pt.) in the EMF + mussel quadrant compared to the Bare and EMF‐only quadrants and increased transiting behaviour (+6%pt.) in the mussel‐only quadrant relative to the EMF + mussel quadrant.

Neither time spent per quadrant nor total distance travelled differed significantly between the elevated EMF and the background EMF quadrants, nor in the mussel or bare quadrants. Based on previous studies (Gill, 2009; Hutchison, Gill, et al., 2020; Labourgade et al., 2024), an attraction to AC EMF exposure might have been expected. A possible explanation for the lack of behavioural change is that previous studies were conducted in mesocosm‐ or field‐based tagging contexts, where electroreception is used in concert with chemical, mechanical and visual signals, which are lacking in a laboratory setting. Altered behavioural responses to anthropogenic EMF may only emerge in more natural conditions where animals are required to continuously integrate multiple sensory cues, potentially interfering with foraging behaviour or predator–prey interactions. Feeding motivation was likely low in the present study, as sharks were fed ad libitum and no food or odour cues were provided to trigger active prey‐search behaviour.

The proportions of inactive and transiting behaviour differed significantly between treatments, although the magnitude of these changes was small (5–6%pt.). Although this behavioural effect was observed, considerable interindividual variation was evident in the data, consistent with previous findings for elasmobranchs (Dhellemmes et al., 2021; Finger et al., 2016; Hermans, Maris, et al., 2025). Individual sharks also did not consistently select the same quadrants across repeated trials (Figure 5 and Figure S1), indicating that quadrant selection was not driven by individual preferences. Previous EMF exposure during embryogenesis in part of the study population cannot be fully excluded as a potential source of individual variation or habituation. However, behavioural responses to the current EMF exposure were limited, being restricted to the proportions of inactive and transiting behaviour, with effects that were only marginally significant. This provides no strong indication that prior exposure substantially influenced the observed patterns, although the possibility cannot be excluded. Whether these subtle behavioural shifts would be amplified under natural conditions with more complex sensory cues, or attenuated against environmental background variability, remains unclear. Overall, while a behavioural effect was detected, its ecological relevance and applicability to field settings cannot yet be resolved.

**FIGURE 5:**
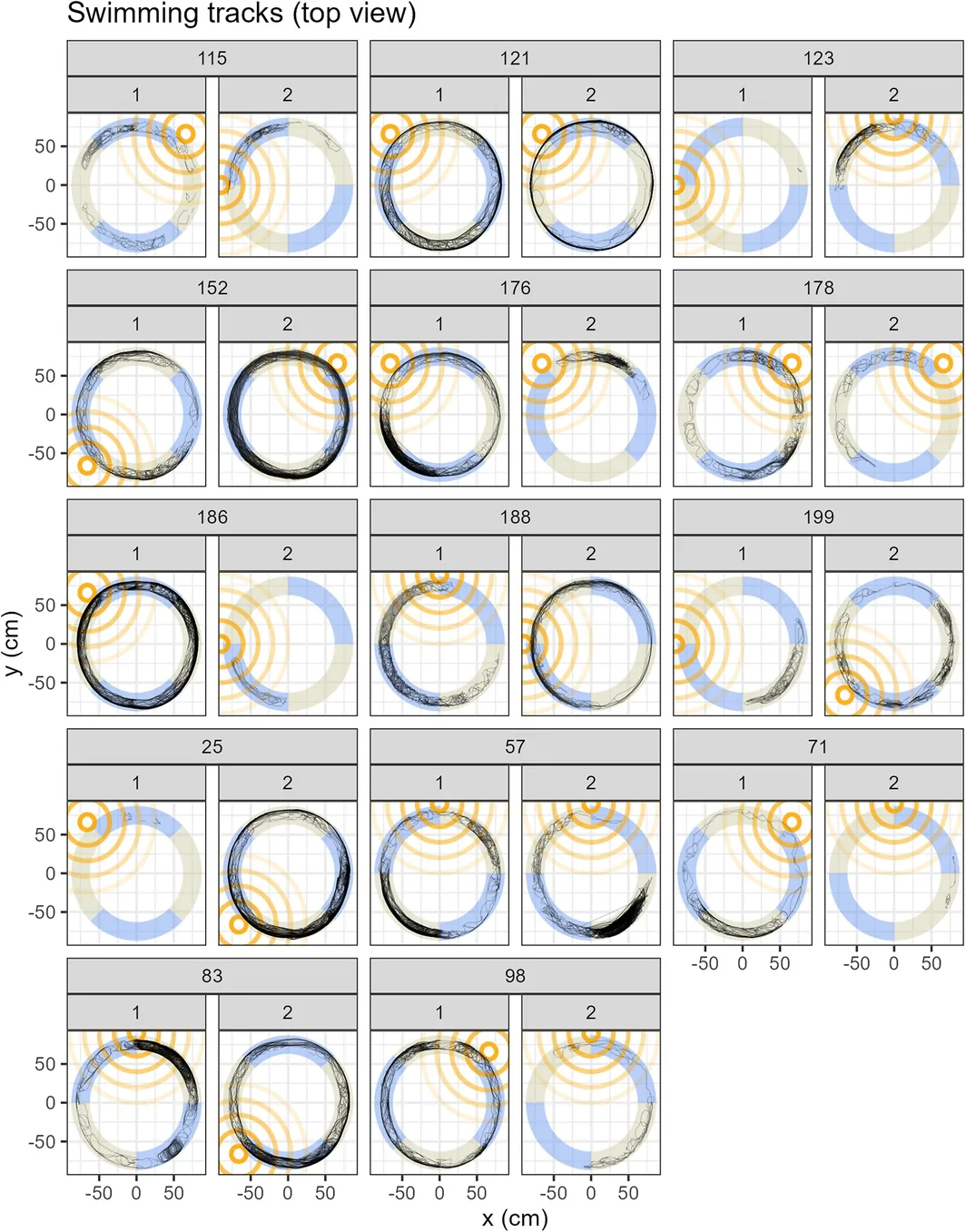
Top view of the swim tracks of all individuals in both their trials. The bar above two tracks indicates the individual, and the bar below indicates whether this was the first or second trial. The shaded background of the tracks indicates the substrate (blue = mussels, beige = bare). The centres of orange circles indicate the position of the EMF coil, note that EMF spreads elliptical rather than circular in reality. In a few trials, the shark did not move much, hence the swimming track is very small.

No significant differences were found in time spent across the four quadrants or behaviour based on the different substrate types provided. Live mussel substrates have been shown to influence fish behaviour, such as providing shelter for European eel (Schwartzbach et al., 2020). Mussel farmers in the Eastern Scheldt report elasmobranch species, including *Dasyatis pastinaca, Mustelus asterias* and *Raja clavata*, during the management of shellfish plots (personal observation). A preference for complex biogenic substrate over non‐complex substrate would seem more in line with natural behaviour as it more closely resembles their natural habitat (Heessen et al., 2015; Martin et al., 2012; Papadopoulo et al., 2023; Pizzolla, 2025). The absence of a substrate effect in the present experiment may reflect the opportunistic nature of *S. canicula*, as it is known to inhabit a wide range of substrates (Ellis et al., 2008), possibly favouring one substrate over another based on incentives such as foraging or mating cues. Sharks did not display mussel‐specific selection, suggesting possible ecological implications that merit further investigation, especially in the light of (shellfish) aquaculture practices.

Even though mussels were included in the housing tank to familiarise the sharks, the absence of preferences for conditions may relate to the use of lab‐reared individuals that lack prior experience with complex natural environments and foraging contexts. Sharks displayed diel activity patterns aligned with light conditions (Figure S2), suggesting that at least part of their behavioural repertoire appears comparable to that of wild individuals and supporting the relevance of their responses to potential field conditions. The covariates that best explained shark behavioural states resulting from the HMM, for example, trial order, light condition and body length (Table S2), can be explained by literature. The observed order effect (Figure S3) is also seen in other studies and may reflect a reduction in novelty or exploratory motivation in individuals over consecutive trials (Dhellemmes et al., 2021; Finger et al., 2016). Alternatively it may indicate a learning or habituation effect influencing movement behaviour across repeated exposures. However, in the present study, the significant effect or trial order is likely largely driven by a transition between a lot of movement and little to no movement in several individuals across trials, which can also be visually observed (Figure 5). Multiple individuals displayed the opposite pattern with more movement occurring during the second trial. Because behavioural changes occurred in both directions, the observed order effect does not appear to reflect a uniform response across individuals. The observed change in activity levels between day and night (Figure S2), being higher at night which is in line with normal behaviour in the wild, and was already discussed above (Papadopoulo et al., 2023). And consistent with previous tagging studies on *S. canicula* (Papadopoulo et al., 2023), larger individuals exhibited reduced swimming activity (Figure S4).

Results from this controlled experiment indicate that AC‐generated EMFs influence behaviour, albeit subtly, but do not influence substrate preference in *S. canicula*. The data do not suggest a barrier resulting from AC EMFs associated with both export cables (running perpendicular to the coastline and transporting electricity from offshore wind farms to shore) and infield cable networks (connecting turbines to the offshore high‐voltage station).

It is important to recognise that laboratory experiments, while controlled, represent a simplification of the natural environment, where substrate preference and behaviour are influenced by multiple interacting sensory cues (Winkler & Murphy, 1973), including chemical, hydrodynamic and social information. Furthermore, the experimental design did not assess several ecologically relevant behaviours that may be influenced by EMF, including complex predator–prey interactions, migration movements and interaction with conspecifics. In this study, the effects of substrate and EMF were explicitly isolated and tested, while other factors potentially influencing behaviour were controlled or standardised to minimise their influence. This experimental design enables a focused assessment of these drivers, including their interaction. It is acknowledged that such controlled conditions do not capture the full complexity of natural systems, and we therefore advocate for integrating these findings with field‐based studies. For example, fine‐meshed field‐based telemetry studies above subsea power cables are needed to investigate habitat use and behavioural responses in ecologically realistic contexts including both natural prey availability and conspecific interactions (Papadopoulo et al., 2023; Reubens et al., 2019). Also, previous studies demonstrate interspecific differences in elasmobranch responses to EMF, highlighting the importance of testing whether the present results generalise to other elasmobranch species. Such a combined approach to capture both laboratory and field dynamics is expected to provide a more comprehensive understanding of *S. canicula* behaviour within an environmental context.

Finally, while robust non‐parametric tests were used to test the habitat preference of the sharks, the statistical power of the study remains limited due to the sample size (*N* = 14). This must be recognised when interpreting the outcomes, as subtle yet potentially relevant patterns may remain undetected. Still, the high p‐values obtained from the statistical tests also show no indication of a trend towards significance.

A number of studies have found comparable subtle or context‐dependent effects of EMF (Gill, 2009; Hermans, Maas, et al., 2025; Hermans, Maris, et al., 2025; Hutchison, Secor, & Gill, 2020; Orr, 2016). However, these minor negative impacts must be weighed against potential positive effects, for example, a refuge benefit that offshore wind farms may provide due to the lack of bottom trawling fisheries. Large areas within OWFs are closed to fishing, the primary source of mortality for elasmobranchs populations, which could result in a net positive conservation outcome even if small behavioural effects occur. Additional data are required to evaluate this balance. Our findings contribute to this perspective by suggesting that EMF itself may result in subtle behavioural changes, but not a barrier for *S. canicula*. Nonetheless, other anthropogenic pressures (e.g., noise, construction disturbance, habitat alteration) are associated with OWFs and warrant parallel investigation.

Looking ahead, offshore power transmission is expected to rely increasingly on direct current (DC) systems, as wind farms are built further offshore and DC transmission minimises energy loss over long distances (Ryndzionek & Sienkiewicz, 2020). Consequently, in the future, the rate of exposure to DC fields will be higher for elasmobranchs and other species (Hermans et al., 2024). The biological relevance of this change is notable, as DC and low‐frequency AC (<20 Hz) fields, comparable to conspecifics and prey, are more readily detected by elasmobranchs than the 50 Hz AC currently used in most grids (Bedore & Kajiura, 2013). Moreover, DC systems, though nominally constant, can generate transient low‐frequency AC fluctuations due to variable power loads, producing a more complex electromagnetic environment. Lastly, the geomagnetic field is a DC field, and sharks are believed to use this for navigation, underscoring the importance of further understanding and studying behavioural responses to DC EMFs.

## 5 CONCLUSIONS

In this controlled laboratory experiment, juvenile *S. canicula* showed no change in time spent nor distance travelled depending on EMF exposure or the presence of mussels as a proxy for complex biogenic substrate. Subtle but significant changes (5–6%pt.) were found in behavioural states. These findings indicate that AC subsea cable fields, at intensities comparable to those found around offshore wind infrastructure, are unlikely to form a hard barrier. Additionally, juvenile sharks did not display mussel‐specific selection, indicating a null preference that may carry ecological relevance for aquaculture managers. While only one minor significant behavioural effect was detected, its ecological relevance and applicability to field settings cannot yet be resolved for juveniles of this benthic shark species. Future research should validate these responses in situ to determine whether the largely absent behavioural effects hold under natural environmental conditions and for different elasmobranch species. In addition, the influence of anthropogenic DC fields on habitat selection and swimming behaviour should be further explored.

## AUTHOR CONTRIBUTIONS

**Lotte Bouwman**: Conceptualisation; data curation; formal analysis; investigation; methodology; project administration; validation; visualisation; writing—original draft preparation; writing—review and editing. **Annemiek Hermans**: Conceptualisation; formal analysis; investigation; methodology; project administration; validation; writing—original draft preparation; writing—review and editing. **Jeroen Hubert**: Formal analysis; investigation; validation; visualisation; writing—original draft preparation; writing—review and editing. **Reindert Nijland**: Supervision; validation; writing—review and editing. **Henrice Jansen**: Supervision; validation; writing—review and editing. **Jildou Schotanus**: Supervision; validation; writing—review and editing. **Hendrik V. Winter**: Supervision; validation; writing—review and editing.

## FUNDING INFORMATION

Funding for this project was granted by the NWO PPS grant ENW PPS‐fonds 2019 (grant number ENPPS.TA.019.005) and PPS‐fonds TKI (LWV22179).

## CONFLICT OF INTEREST STATEMENT

The authors declare no conflict of interest.

## Supporting information


**Table S1.** Model selection to determine optimal number of behavioural states using Hidden Markov Models to explain shark behaviour during the experiment. The AIC is the Akaike Information Criterion, and the lowest AIC refers to the best model [*]. In the case, a 3‐state model was supported best.
**Table S2.** Model selection to determine the covariates for the best fit to explain shark behaviour during the experiment. The null‐model, without covariates is indicated by [~1]. The AIC is the Akaike Information Criterion, and the lowest AIC indicates the best model [*].
**Table S3.** Overview of all trials, dates and entry‐ and EMF coil positions.
**Figure S1.** State distribution for each of the 14 sharks in each quadrant. The numbers on the right indicate the individual shark ID, overarching trial 1 and 2 representing the two trials of each shark. The numbers in the bar represent the amount of time spent in that quadrant (XY positions recorded). The asterisks indicates the release position of the shark at the start of the acclimation (3 h prior to the behavioural monitoring).
**Figure S2.** Stationary state probability of the behavioural states during light‐time and dark‐time. Sharks were significantly more active during dark‐time, both locally active and transiting.
**Figure S3.** Stationary state probability during the first and second trial of each individual. Sharks were significantly less active in the second trial.
**Figure S4.** Stationary state probability over trial body length of the sharks, shaded area indicates the 95% confidence interval. Larger individuals spent more time being inactive.

## Data Availability

The data that support the findings of this study are available from the corresponding author upon reasonable request.
