# Supplementary material for: Shark preferences: Only subtle effects of electromagnetic field exposure and substrate type on small‐spotted catshark (Scyliorhinus canicula) behaviour
Source: J Fish Biol. 2026 Jul 28;109(4):2166–78. doi: 10.1111/jfb.70583 (PMC13675498; doi:10.1111/jfb.70583)
Supplement: Supplementary file 1 [file JFB-109-2166-s001.docx]

Supplementary materials

Table S1: Model selection to determine optimal number of behavioural states using Hidden Markov Models to explain shark behaviour during the experiment. The AIC is the Akaike Information Criterion, and the lowest AIC refers to the best model [*]. In the case, a 3-state model was supported best.

| **Number of states** | **AIC** |
| --- | --- |
| 3* | 1196546 |
| 2 | 1732541 |
| 1 | 3527098 |

Table S2: Model selection to determine the covariates for the best fit to explain shark behaviour during the experiment. The null-model, without covariates is indicated by [~1]. The AIC is the Akaike Information Criterion, and the lowest AIC indicates the best model [*].

| **#** | **Covariates** | **AIC** |
| --- | --- | --- |
| ***1** | Light condition + Quadrant + Order + Body length | 1992768 |
| **2** | Light condition + Quadrant + Order | 1193349 |
| **3** | Light condition + Quadrant | 1194237 |
| **4** | Light condition + Order | 1194479 |
| **5** | Light condition | 1195355 |
| **6** | Order | 1195474 |
| **7** | Quadrant | 1195493 |
| **8** | ~1 | 1196546 |
| **9** | Light condition + Quadrant + Body length | 1200506 |
| **10** | Light condition + Body length | 1566701 |
| **11** | Body length | 1567758 |

Table S3: Overview of all trials, dates and entry- and EMF coil positions.

|  | Trial 1 | | | Trial 2 | | |
| --- | --- | --- | --- | --- | --- | --- |
| Shark ID | Date | Position Entry | Position coil | Date | Position Entry | Position coil |
| 199 | 20-5-2025 | 3 | 2 | 12-6-2025 | 2 | 2 |
| 25 | 21-5-2025 | 6 | 3 | 27-5-2025 | 4 | 3 |
| 121 | 23-5-2025 | 6 | 3 | 23-6-2025 | 2 | 3 |
| 176 | 24-5-2025 | 1 | 3 | 26-6-2025 | 7 | 3 |
| 115 | 26-5-2025 | 2 | 5 | 17-6-2025 | 4 | 5 |
| 178 | 2-6-2025 | 1 | 5 | 10-6-2025 | 7 | 5 |
| 83 | 28-5-2025 | 2 | 4 | 9-6-2025 | 3 | 4 |
| 188 | 29-5-2025 | 6 | 4 | 20-6-2025 | 3 | 4 |
| 98 | 3-6-2025 | 1 | 5 | 11-6-2025 | 3 | 5 |
| 186 | 4-6-2025 | 8 | 3 | 16-6-2025 | 4 | 3 |
| 57 | 5-6-2025 | 5 | 4 | 13-6-2025 | 8 | 4 |
| 123 | 8-6-2025 | 8 | 2 | 19-6-2025 | 2 | 2 |
| 152 | 22-5-2025 | 5 | 1 | 25-6-2025 | 7 | 1 |
| 71 | 24-6-2025 | 2 | 5 | 27-6-2025 | 4 | 5 |

Figure S1: State distribution for each of the 14 sharks in each quadrant. The numbers on the right indicate the individual shark ID, overarching trial 1 and 2 representing the two trials of each shark. The numbers in the bar represent the amount of time spent in that quadrant (XY positions recorded). The asterisks indicates the release position of the shark at the start of the acclimation (3 hours prior to the behavioural monitoring).

Figure S2: Stationary state probability of the behavioural states during light-time and dark-time. Sharks were significantly more active during dark-time, both locally active and transiting.

Figure S3: Stationary state probability during the first and second trial of each individual. Sharks were significantly less active in the second trial.

Figure S4: Stationarity state probability over trial body length of the sharks, shaded area indicates the 95% confidence interval. Larger individuals spent more time being inactive.
